# Macrophage activation syndrome revealing systemic lupus erythematosus: about 4 cases

**DOI:** 10.1186/1546-0096-12-S1-P217

**Published:** 2014-09-17

**Authors:** Kenza Bouayed, Alexandre Belot, K Bouayed, M El Bouz, I Chahid, N Mikou, E Charrier, S Viel, C Malcus, T Walzer, A Belot

**Affiliations:** 1 CHU Ibn Rochd, Casablanca, Morocco; 2 Pediatric Rheumatology Unit, Hôpital Femme Mère Enfant, hospices civils de Lyon, Lyon, France

## Introduction

The macrophage activation syndrome (MAS) in its secondary form is known to complicate rheumatic diseases as systemic lupus erythematous (SLE) but rarely realizes a form of revelation.

## Objectives

The aim of our study is to report unrecognized MAS manifestation as an initial symptom of SLE and describe both clinical and immunological characteristics in 4 patients.

## Methods

Cases Report.

## Results

We report on four girls, aged of 13, 13, 10 and 7 years old, initially admitted at our university hospital for prolonged fever. Main infectious disease causes were ruled out and a diagnosis of MAS was made according to the criteria of HLH 2004. During the follow up, all patients presented with SLE symptoms and fulfilled the modified ARA criteria. Glomerular nephropathy was clinically diagnosed in our four patients and was confirmed on biopsy, [Pt 1]: class I, [Pt 2]: class V, [Pt 3]: class IV & V, [Pt 4]: class IV. All of them had positive antinuclear and anti-dsDNA antibodies associated to low complement levels. Patients were treated by steroids pulse and DMARDs. The evolution was satisfactory with a follow up of 4-28 months. Three of our patients underwent an immunological assessment. Interestingly, 6 months after the MAS, [Pt 1] was still presenting an increase of activated HLA-DR+ CD3 T cells (39.4%) with very low NK cells level. CD4+CD25+CD127- Treg cells were also decreased. 1 month after initial MAS, [Pt2] presented with activated CD8 T cells in agreement with persisting MAS –related activation and Tregs were reduced. NK cells were also decreased. Perforin expression was normal. Pt 3 immunophenotyping was in normal range after 28 months.

## Conclusion

The diagnosis of MAS is challenging in the context of SLE because many clinical and biological manifestations are overlapping. It is even harder when it inaugurates lupus. Therefore, it is crucial to consider both MAS and SLE diagnosis in the contest of multi- visceral manifestations with unexplained fever. Diagnosis rely on biological assessment of MAS (hypertriglyceridemia , hyperferritinemia , cytolysis and pancytopenia) associated with the presence of a hemophagocytes in bone aspiration. Early targeted treatment is mandatory and overall prognosis is favorable. As two unrelated patients have shared common clinical and immunological features, some inherited factors may bridge SLE and MAS pathogenesis. Whole exome sequencing of these patients may highlight new forms of mendelian SLE.

## Disclosure of interest

None declared.

